# Southward autumn migration of waterfowl facilitates cross-continental transmission of the highly pathogenic avian influenza H5N1 virus

**DOI:** 10.1038/srep30262

**Published:** 2016-08-10

**Authors:** Yanjie Xu, Peng Gong, Ben Wielstra, Yali Si

**Affiliations:** 1 Ministry of Education Key Laboratory for Earth System Modeling, and Center for Earth System Science, Tsinghua University, Beijing, China; 2 Joint Center for Global Change Studies, Beijing 100875, China; 3 Department of Animal and Plant Sciences, University of Sheffield, S10 2TN Sheffield, UK; 4 Naturalis Biodiversity Center, P.O. Box 9517, 2300 RA Leiden, The Netherlands; 5 Resource Ecology Group, Wageningen University, Droevendaalsesteeg 3a, 6708 PB Wageningen, The Netherlands

## Abstract

The highly pathogenic avian influenza subtype H5N1 (HPAI H5N1) is a worldwide zoonotic infectious disease, threatening humans, poultry and wild birds. The role of wild birds in the spread of HPAI H5N1 has previously been investigated by comparing disease spread patterns with bird migration routes. However, the different roles that the southward autumn and northward spring migration might play in virus transmission have hardly been explored. Using direction analysis, we analyze HPAI H5N1 transmission directions and angular concentration of currently circulating viral clades, and compare these with waterfowl seasonal migration directions along major waterfowl flyways. Out of 22 HPAI H5N1 transmission directions, 18 had both a southward direction and a relatively high concentration. Differences between disease transmission and waterfowl migration directions were significantly smaller for autumn than for spring migration. The four northward transmission directions were found along Asian flyways, where the initial epicenter of the virus was located. We suggest waterfowl first picked up the virus from East Asia, then brought it to the north via spring migration, and then spread it to other parts of world mainly by autumn migration. We emphasize waterfowl autumn migration plays a relatively important role in HPAI H5N1 transmission compared to spring migration.

Wild birds are considered to be the natural reservoir for avian influenza viruses, and a particularly high prevalence rate has been observed in waterfowl, including Anseriformes and Charadriiformes^1^. The highly pathogenic avian influenza subtype H5N1 (HPAI H5N1) is a worldwide zoonotic disease, threatening humans, poultry and wildlife. Since the first detection of HPAI H5N1 in farm geese in China in 1996, up to March 2015 HPAI H5N1 has been found in 69 countries in Asia, Europe, Africa, and the Americas (http://empres-i.fao.org/ empres-i/home). While poultry are the primary reservoir of highly pathogenic avian influenza viruses, wild waterfowl can act as secondary transmitters^2^,^3^. Hence, the HPAI H5N1 virus can spread over long distances, not only via trade in poultry and wild-caught birds, but also via the natural movements of wild birds^4^. Initially, wild birds were most likely infected with HPAI H5N1 via contact with infected poultry in regions where poultry density is high^5^ (see Fig. 1). Subsequently, asymptomatic infection, as reported in some duck species^3^,^6^, and the ability of the virus to survive in wild bird habitats without a host for over a month^7^, would make it possible for wild birds to disperse HPAI H5N1 across large distances and time spans, without the direct involvement of poultry^8^.

The first considerable HPAI H5N1 outbreak in migratory waterfowl was recorded at Qinghai Lake in April 2005 (clade 2.2 according to the WHO naming system^9^) and resulted in mass die-offs for bar-headed geese^10^,^11^. Since then, HPAI H5N1 viruses closely related to the Qinghai-like clade 2.2 continued to be isolated along the Eurasian migration flyways and resulted in mortality of millions of wild and domestic birds in Asia, Europe, and Africa^4^,^12^,^13^. Another novel clade, clade 2.3.2, was recorded in Qinghai Lake in 2009 and has caused the death of over two hundred wild birds^5^,^14^. Clade 2.3.2.1 was firstly recorded in whooper swan (*Cygnus cygnus*) in Japan in 2008^5^,^14^ and has been found in wild waterfowl in Mongolia, Russia, Korea, and Japan during 2009–2011^13^,^15^,^16^, and more recently in China in 2015^17^. Other outbreaks of the HPAI H5 lineages H5N8 and H5N2, are considered to have been spread from Asia to Europe and North America via waterfowl migration^18^,^19^. These studies highlight that wild birds could carry and spread avian influenza viruses along their flyways.

The role of migratory birds in the spread of HPAI H5N1 has been further investigated by comparing disease spread patterns with bird migration patterns. Six HPAI H5N1 outbreak patterns were found to be spatio-temporally associated with the seasonal migration of waterfowl using a space-time clustering analysis^20^. By combining space-time and phylogenetic approaches, Liang *et al*.^21^ reported possible spread routes for HPAI H5N1 that coincided well with four major flyways. Gaidet *et al*.^22^ analyzed a large-scale dataset of wildfowl movements over HPAI H5N1 infected regions in Eurasia and Africa and suggested potential long-distance spread of HPAI H5N1 by migratory birds could occur. According to the relationship between viral transmission and waterfowl migration networks along the East Asian-Australasian and Central Asian Flyway, waterfowl migration was considered as the most likely factor explaining the geographic transmission of HPAI H5N1 viruses (clade 2.3.2)^23^. Siberia, which intersects with multiple flyways and is considered as one the most important breeding sites for migratory waterfowl, played a vital role in the global HPAI H5N1 transmission, with the highest emigration and second highest immigration rate^24^.

Next to the general match between HPAI H5N1 outbreaks and waterfowl migration patterns, several researchers have detected different levels of disease outbreak risk during the distinct waterfowl spring and autumn migration periods. Anatidae were found likely to spread the HPAI H5N1 virus along their autumn migration routes from Russia and Kazakhstan to the Black Sea region^25^. Bourouiba *et al*.^26^ found that repeated outbreaks of HPAI H5N1 at stopovers caused more mortalities during autumn migration than during spring migration. Analysis of the distance between disease outbreak sites in poultry and the closest migratory waterfowl sites in Romania indicated that HPAI H5N1 infections of poultry might occur via exposure to migratory waterfowl during autumn^27^. Yet, core flyway corridors of wild ducks tracked along the East Asian Flyway during spring migration were not spatio-temporally connected to sites of reported outbreaks^28^. While these studies suggest potential seasonal variation in waterfowl migration in the spread of HPAI H5N1, whether there is a difference in importance between autumn and spring migration still has to be investigated. Yet, such information is pertinent for understanding the disease transmission mechanisms and is crucial for targeted prevention efforts.

Direction analysis is an efficient way to accurately quantify disease transmission direction and angular concentration. Direction analysis can be used to compare disease spread directions with the distinct seasonal migration directions of waterfowl. However, only few studies adopted direction analysis for determining the spread of HPAI H5N1 and they mostly were restricted to a local or a regional scale. For example, the directions of HPAI H5N1 transmission in Bangladesh^29^,^30^ and Romania^31^ were quantified and compared with local movements of wild birds and poultry. To get a further insight into the spatial-temporal relationships between continental waterfowl migration and HPAI H5N1 transmission, it is necessary to carry out direction analysis at the continental scale.

In this paper we use HPAI H5N1 viral clades as input for direction analysis. The HPAI H5N1 transmission directions and angular concentrations are then compared with waterfowl seasonal migration directions along major waterfowl flyways. We are particularly interested in testing how waterfowl seasonal migration facilitates the transmission of HPAI H5N1. Considering latitudinal migration of waterfowl during their annual life cycle (travelling to the high-latitude breeding grounds in spring and returning to the low-latitude wintering sites in autumn), if disease transmission directions along each flyway point in a southward direction, we postulate that waterfowl autumn migration facilitates transmission, whereas if directions point in a northward direction we postulate that spring migration facilitates transmission. This study is the first to systematically quantify the transmission directions and concentration of HPAI H5N1 viruses along the major flyways and compare these to waterfowl seasonal migration directions. Our findings contribute to global avian influenza surveillance and control.

## Results

### Spatio-temporal dimensions of HPAI H5N1 viral clades

**Figure 1:**
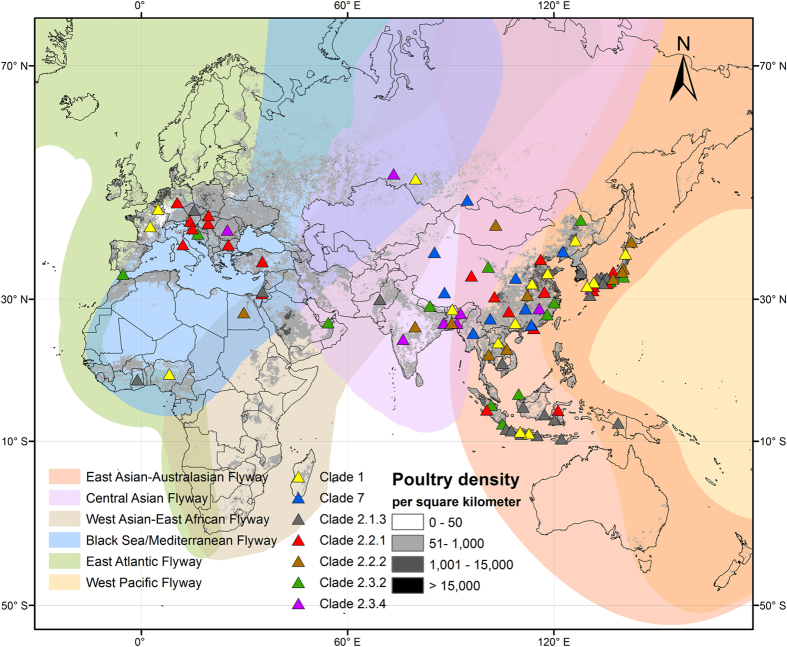
Distribution of HPAI H5N1 genetic clades and poultry density. One clade sampled along the Mississippi Americas Flyway was omitted from the map due to the small sample size. The map was produced using ArcGIS Desktop 10.3 (www.esri.com).

The sample sites of different clades are shown in Fig. 1. A total of 433 sample sites for clades 1 (n = 34), 2.1.3 (n = 54), 2.2.1 (n = 96), 2.2.2 (n = 20), 2.3.2 (n = 130), 2.3.4 (n = 78), and 7 (n = 21) were obtained for subsequent analysis. All these clades were intensively sampled in East China, Japan, Southeast Asian countries (Indonesia, Malaysia, Cambodia, Thailand, Laos, Vietnam, and Myanmar), Southern Asian countries (India, Bangladesh, Bhutan, Nepal, and Pakistan) and Egypt. All clades except clade 2.2.2 were recovered in Russia. Only clades 2.2.1 and 2.2.2 were found in Mongolia. Clades 1, 2.1.3, and 2.2.1 were found in Africa in Nigeria and Ghana. In Europe, clades 1, 2.1.3, 2.2.1, 2.3.2, and 2.3.4 were sampled from Italy, Bulgaria, Romania, Croatia, Slovenia, Austria, Hungary, Slovakia, Czech Republic, France, Germany, and Belgium.

All seven clades were sampled along the East Asian-Australasian, Central Asian, West Asian-East African, Black Sea/Mediterranean, and West Pacific Flyways. Along the East Atlantic Flyway, clades 1, 2.2.1, 7 (2 sites), 2.1.3 (2 sites), 2.3.2 (2 sites) were sampled. Along the West Asian-East African and Black Sea/Mediterranean Flyways, clade 2.2.2 was only sampled in a single site. Along the Black Sea/Mediterranean Flyway clade 7 was only sampled in a single site. Along the Mississippi Americas Flyway only one clade (2.3.2) was sampled in Washington in 2014; the sample size for this flyway is too small to be included in the direction analysis.

In regards to the temporal dimension, all the seven clades were sampled since 2004, and most of them were firstly found along the East Asian-Australasian Flyway (Table 1). The longevity of each clade is: clade 1 (2004–2012), clade 7 (2004–2015), clade 2.1.3 (2004–2015), clade 2.2.1 (2004–2015), clade 2.2.2 (2004–2013), clade 2.3.2 (2004–2015), and clade 2.3.4 (2004–2015). The number of years each clade was sampled along different flyways is: clade 1 (2–9 years), clade 7 (0–7 years), clade 2.1.3 (1–9 years), clade 2.2.1 (2–11 years), clade 2.2.2 (0–5 years), clade 2.3.2 (1–11 years), and clade 2.3.4 (0–11 years).

### Directions of HPAI H5N1 transmission and waterfowl seasonal migration

Table 2 show the transmission direction and angular concentration of HPAI H5N1 genetic clades. Table 3 shows the waterfowl seasonal migration directions and concentrations in the four infected flyways. For all directions, 90 degree is due north and 0 degree is due east. We define directions within 0–180 degree as northward directions and 180–360 degree as southward directions. In total 22 directions with significant angular concentrations (*P* ≤ 0.01) were found along the East Asian-Australasian (2 northward and 3 southward), Central Asian (5 southward), West Asian-East African (1 northward and 3 southward), Black Sea/Mediterranean (3 southward), East Atlantic (1 southward) and West Pacific (1 northward and 3 southward) Flyways (Tables 2 and 3). Three flyways with either large or small sample size yielded both southward and northward directions, indicating there was no correlation between sample size and the detection of bi-directional patterns. The mean concentration for southward disease transmission directions (0.41) was higher than for northward directions (0.25).

**Figure 2:**
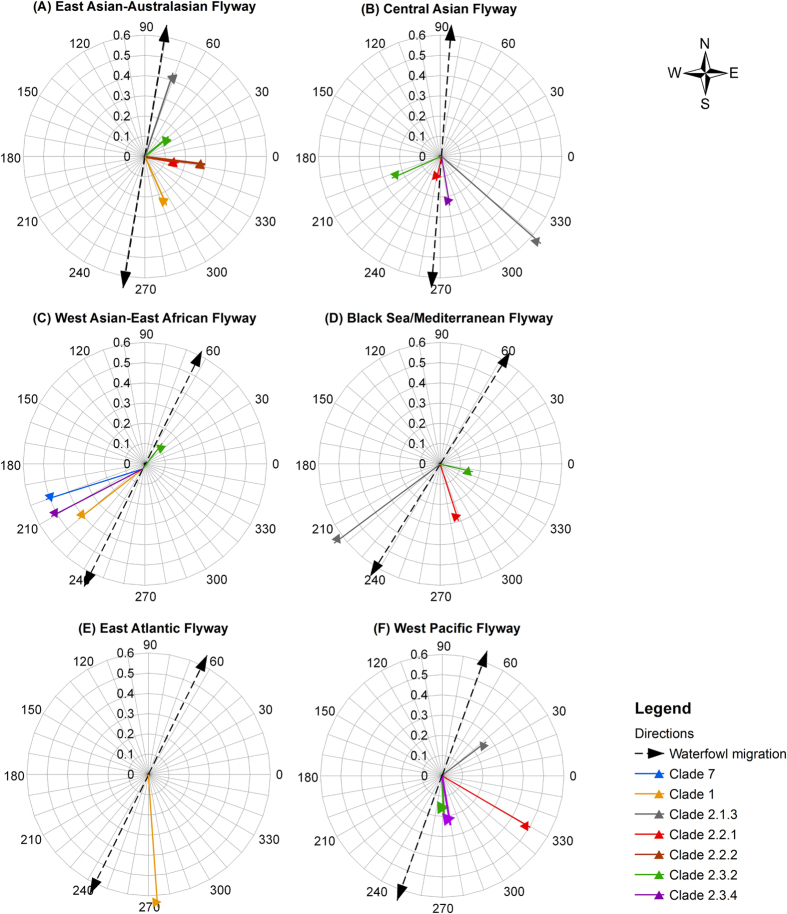
Directions of HPAI H5N1 transmission and waterfowl seasonal migration. The graphs represents calculated direction (degree) for HPAI H5N1 genetic clades for the six main infected flyways; the length of the arrow (see axis) represents the angular concentration of each direction; all the directions displayed are statistically significant (*P* ≤ 0.01) in direction tests; the concentrations for waterfowl migration directions are all greater than 0.6.

**Figure 3:**
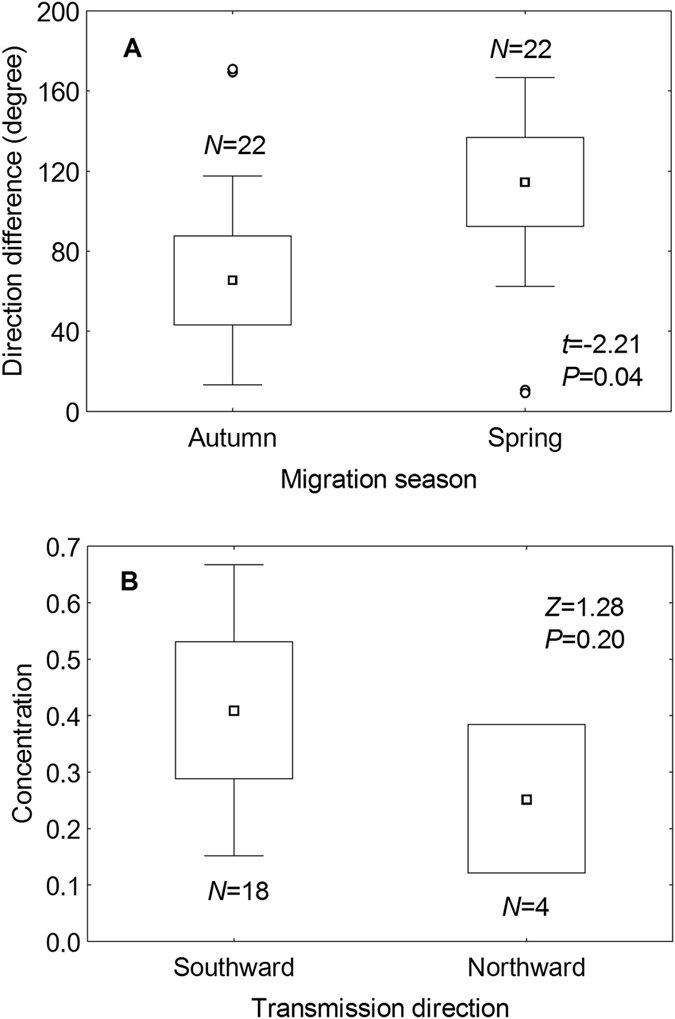
Differences between HPAI H5N1 transmission and waterfowl seasonal migration. The boxplot compares differences in (**A**) direction differences with waterfowl autumn and spring migration directions; (**B**) the concentrations of southward and northward disease transmission directions. The boxes represent the standard error range; the bars cover the standard deviation range; the midpoint is the mean of each group; and the circles show outliers.

Most (18 out of 22) of the calculated HPAI H5N1 transmission directions overlapping with the major flyways were oriented southward and matched better with autumn than with spring waterfowl migration direction (Fig. 2). The differences between the disease transmission direction and waterfowl migration direction along the six flyways were normally distributed (Kolmogorov-Smirnov Test, *N* = 44, *P* = 0.16). The HPAI H5N1 transmission directions showed a significantly smaller difference with autumn migration directions than with spring migration directions (Paired-Samples T Test, *t* = −2.21, *N*_*1*_ = *N*_*2*_ = 22, *P* = 0.04) along these flyways (Fig. 3A). The concentrations of significant transmission directions were not normally distributed (Kolmogorov-Smirnov Test, *N* = 22, *P* = 0.01). Although the mean concentration for southward transmission directions was higher than for northward ones, they did not significantly differ from each other (Mann-Whitney U Test, *Z* = 1.28, *N*_*1*_ = 18, *N*_*2*_ = *4*, *P* = 0.20) (Fig. 3B).

## Discussion

This study quantified the HPAI H5N1 spread directions derived from genetic clades and compared them with waterfowl seasonal migration directions. Eighteen out of 22 HPAI H5N1 transmission directions were oriented southwards, in close proximity to waterfowl autumn migration directions. Moreover, the concentrations for southward disease transmission directions were higher than that for northward directions. Our findings emphasize a relatively important role of waterfowl autumn migration in facilitating HPAI H5N1 cross-continental transmission compared to spring migration.

Possible explanations for the close proximity of HPAI H5N1 transmission directions to waterfowl southward autumn migration are as follows. Firstly, southward autumn migration includes juveniles born the previous breeding season, meaning the population size is larger in autumn than in spring. Due to the positive relationship between virus prevalence and migratory waterfowl density^32^, a higher migratory waterfowl density in autumn may facilitate an increased avian influenza prevalence^33^. Secondly, the population structure with more vulnerable juveniles leads to a higher disease infection rate during autumn migration. Olsen *et al*.^34^ indicated a higher low pathogenic avian influenza (LPAI) virus prevalence in juveniles in early autumn. Waterfowl with prior exposure to LPAI show immunity to HPAI H5N1^35^. Juveniles are immunologically naive in comparison to adults and are therefore more prone to being infected by and shedding avian influenza viruses^33^,^36^. Thirdly, autumn migration is less synchronized and more flexible than spring migration. Various species migrate north in spring and gather in their northern breeding grounds creating a virus pool^21^,^24^. After breeding, they migrate southwards to their wintering sites using different migration routes, which could cause a redistribution of avian influenza viruses and contribute to the survival of the virus across a wide geographic range. This process would also help transmit the virus to the resident avian population in the wintering sites of waterfowl^37^.

HPAI H5N1 southward transmission has been reported in previous studies using phylogenetic analysis in combination with trajectory analysis of cross-continent viral movements. Liang *et al*.^21^ found that the HPAI H5N1 virus spread in a southward direction along the West Asian-East African and Black Sea/Mediterranean Flyways using Siberian breeding land as a hinge area. Li *et al*.^38^ observed a “high-to-low latitude” transmission pattern coinciding with the autumn migration of waterfowl along the Central Asian and East Asian-Australasian Flyways. As indicated by Newman *et al*.^39^, along the Central Asian Flyway, the spatial pattern of viral evolution derived from phylogeographic mapping shows southward movements, coinciding with the autumn migration of waterfowl. However, previous studies^36^,^37^ that linked outbreak data with individual bird satellite tracking data also reported northward transmission directions. Disease directions identified from outbreak data tend to overlook the genetic similarity of these outbreaks and might lead to less rigorous conclusions.

We found both northward and southward HPAI H5N1 spreads along the East Asian-Australasian, West Asian-East African, and West Pacific Flyways. This reflects a unique role of East Asia in HPAI H5N1 transmission. Southern China has been hypothesized as the initial epicenter of the epidemic as the HPAI H5N1 virus was first discovered there in poultry^40^. Clade 2.3.2 was suggested to be transmitted from southern Asia to Mongolia by waterfowl on their way to their northern breeding grounds^15^, which is consistent with the northward transmission directions of clade 2.3.2 in our results. This suggests that migratory birds initially got infected with the HPAI H5N1 virus in East Asia, via contact with poultry, and then spread it northwards during spring migration^21^,^24^. The higher number of southwards disease spread directions suggest that after the virus was brought to north, waterfowl circulated the virus to other parts of world mainly via southward autumn migration. Although poultry transportation cannot be ruled out as a vector in the identified patterns of virus transmission, it would not be expected to leave nearly unidirectional transmission patterns. Furthermore, a similar pattern of disease spread has been reported in the newly emerged HPAI H5N8 virus^17^,^41^,^42^. The H5N8 virus was initially found in China in 2013, was hypothesized to next have been brought to Siberia by waterfowl via northwards spring migration in 2014, and subsequently in the same year spread to Europe, North America and East Asia along different flyways via southwards autumn migration^17^,^41^,^42^.

The continental relay transmission of avian influenza is complex, involving successively infected birds, not necessarily of the same species, but particularly those that share the same habitat^22^. HPAI H5N1 transmission might be aggravated by a large density and diversity of waterfowl with asynchronous timing of arrival and departure^41^. The avian influenza virus can survive longer in cold environments. For example, it can retain infectivity in lake water for more than 30 days at 0 °C, and in faeces for more than 30 days at 4 °C^7^. High HPAI H5N1 infection rates in wild birds might be related to waterfowl movements and congregation along the 0 °C isotherm^42^. This facilitates disease transmission via a “relay race”, i.e., the virus in water body is taken up by different migratory bird populations at different times. The pathobiology of the HPAI H5N1 virus infection varies between different waterfowl species, from fatal to asymptomatic^43^. Such characteristic can cause a mismatch between the timing of HPAI H5N1 outbreaks and waterfowl presence. The interaction between waterfowl and domestic poultry in shared habitat also facilitates HPAI H5N1 transmission^8^,^44^,^45^. This makes it possible for wildfowl to spread HPAI H5N1 over more than one annual migration cycle. Previous studies directly comparing disease outbreak patterns with individual bird migration patterns^28^,^46^,^47^,^48^,^49^ tended to overlook such relay and delay effects.

As a way forward, we analyzed disease transmission directions based on viral clades and calculated waterfowl spring and autumn migration directions separately for each flyway, based on the actual distribution of wetlands. The migration directions generated in this way are considered to encompass the total waterfowl population migrating along this flyway, in contrast to the migration pattern derived from satellite tracking data of just a handful of individuals. However, our findings should be further validated by using a large number of individual bird tracks. In this respect, birds that conduct exploratory movements that cross over flyways^50^ should be considered. Waterfowl tracking data recording information on the age of the birds involved can be used to integrate the age effect on HPAI H5N1 transmission.

In conclusion, we highlight a relatively important role of southward waterfowl migration in cross-continental HPAI H5N1 transmission compared to spring migration, especially after the virus had been introduced to the north. This knowledge facilitates further epidemiological investigations in terms of explaining and predicting the avian influenza transmission in an ecological context. On top of the routine monitoring of waterfowl seasonal migration, we suggest to strengthen the surveillance and control at waterfowl breeding sites, stopover sites during autumn migration, and wintering sites. Siberia is not only the major hub in the global HPAI H5N1 transmission network^24^, but also the common starting point of autumn migration for the different flyways, so we recommend future investigation into disease transmission at the northern breeding sites. Inadequate understanding of the migration ecology of most avian species may limit our ability to model and predict disease transmission^51^,^52^. Therefore, detailed bird migration data are needed to further validate the findings of this study.

## Methods

### Data

We obtained a total of 2867 hemagglutinin (HA) sequences with at least 1600 nucleotides, sampled from avian hosts during 2004–2015, from the Epiflu Database in GISAID (www.gisaid.org). We used the NIAID Influenza Research Database (IRD)^53^ at http://www.fludb.org, which uses phylogenetic analysis, to classify HA sequences to clade according to the WHO classification scheme^9^. The information of each HPAI H5N1 sequence includes sequence name, clade name, host species, location and year of sampling. The locations of the sequences are generally available at a country level, except for several countries such as China and Russia for which province- or city-level data are available. The geographic centroid of the given country or province was used to describe the geographic location of each sequence. Sequences sampled in the same location and year were merged into a single sample site. We excluded clades that have not been detected since 2008 and focused on those seven clades marked as currently circulating by Smith *et al*.^54^, namely clade 1, clade 2.1.3, clade 2.2.1, clade 2.2.2, clade 2.3.2, clade 2.3.4 and clade 7.

The major waterfowl flyways identified in the global monitoring program of Wetland International and the Level 1 Global Lakes and Wetlands Database (GLWD-1)^55^ were used for further direction analysis of waterfowl seasonal migration. Gridded poultry density was obtained from the Global Poultry Density (2005) Dataset in FAO GeoNetwork (www.fao.org/geonetwork/).

### Direction analysis for HPAI H5N1 transmission

The direction method^56^ was applied to calculate the direction and concentration of HPAI H5N1 transmission. Whether the spread of disease tended to be in a particular direction, and the concentration of the direction, were tested with the direction statistic. The null hypothesis for the direction statistic is that the transmission directions across time are dissimilar to each other. The test statistic is a vector and consists of both directions and angular concentrations. The direction is the mean direction of the chains of HPAI H5N1 transmission connected from one HPAI H5N1 clade sample site to another. We used the relative time-connection matrix, which assumed each sample site is related to all sites after it. The concentration is the reciprocal of angular variance of a HPAI H5N1 transmission chain, ranging from 0 to 1; the larger the concentration is, the more the subsequent clade sample sites point to the same direction. The significance of the test statistic was estimated by 999 Monte Carlo simulations. An angular concentration is significant at a 0.01 level when it ranks in the top ten among the random simulated concentrations.

HPAI H5N1 viral clades overlapping with a specific flyway were utilized as the input for the direction statistics. The geographic coordinates of viral clades were transformed into projected x, y coordinates under Mercator (world) projection, which minimizes the distortion on angels. We estimated the transmission directions and angular concentrations of HPAI H5N1 viral clades across each flyway. The direction statistic was calculated with ClusterSeer version 2.5 (www.biomedware.com).

### Waterfowl seasonal migration directions

Waterfowl mostly undertake latitudinal seasonal migration, using wetlands as their stopover sites. We therefore consider the wetlands inside the range of major flyways as potential stopover sites for waterfowl. The GLWD-1 database consists of 3,067 large lakes with more than 50 km^2^ area and 654 large reservoirs with more than 0.5 km^3^ storage capacity from all over the world^55^. The geometric centers of each wetland site were transformed into projected x, y coordinates under world Mercator projection. According to HPAI H5N1 outbreak regions, a total of 1,416 wetlands within a latitude from −10 to 90 and a longitude from −12 to 180 were selected for direction statistics. The temporal information of each wetland was defined based on their latitudes. Generally, waterfowl migrate south during autumn migration, so the lower the latitude of a wetland is, the later waterfowl might encounter it during their migration. For spring migration the reverse applies. Waterfowl seasonal migration directions and concentrations were calculated by direction statistics across different flyways.

### Comparison between HPAI H5N1 transmission and waterfowl seasonal migration directions

HPAI H5N1 transmission directions and waterfowl migration directions with a significant level of *P* ≤ 0.01 were maintained for further analysis. The difference between disease transmission and waterfowl migration directions was the absolute value of each disease transmission direction in a flyway minus the waterfowl migration direction in the same flyway. Therefore, each disease transmission direction generated two differences, one for waterfowl autumn migration direction (*dA*) and another for waterfowl spring migration direction (*dS*). We then evaluated the normality of these differences with the Kolmogorov-Smirnov test. If they were normally distributed, a Paired-Samples T Test would be conducted to test the difference between *dA* and *dS*. If not, a Wilcoxon Matched Pairs Test would be conducted.

We also tested whether there is a significant difference between the concentration of northward (0–180 degree) and southward directions (180–360 degree). The normality of concentrations was tested with a Kolmogorov-Smirnov test; we applied Independent-Samples T Test for normally distributed data and otherwise a Mann Whitney U Test. All statistical analyses were run in Statistica 7 (http://www.statsoft.com/).

## Additional Information

**How to cite this article**: Xu, Y. *et al*. Southward autumn migration of waterfowl facilitates cross-continental transmission of the highly pathogenic avian influenza H5N1 virus. *Sci. Rep.* **6**, 30262; doi: 10.1038/srep30262 (2016).

## Figures and Tables

**Table 1:** Number of sequences sampled per HPAI H5N1 clade in different years along main waterfowl flyways.

Clade	Flyway	2004	2005	2006	2007	2008	2009	2010	2011	2012	2013	2014	2015
1	AAF	60	79	4	15	23	1	11	4	2			
	CAF		1							2			
	WEF		1	6	6			1	1				
	BMF		1	13	11			1	1				
	EAF			7	5								
	WPF	2				1			2				
7	AAF	5	3	5		20		1	1	4			
	CAF		2	2				4			1		
	WEF		2				3	1					1
	BMF						3	1					1
	WPF			1					1				
2.1.3	AAF	4	9	22	75	15	18	20	9	1		1	
	CAF			3	3	5			1	1	1		
	WEF			6	3	5							
	BMF			7									1
	EAF			2									1
	WPF			3				6	7				
2.2.1	AAF	3	4	6	23	4	28	22	31	8	18	4	
	CAF		1	2	2	5	11	7	1	17	2		2
	WEF			31	70	58	30	30	54	7	1		2
	BMF		2	55	70	55	30	30	54	7	1		7
	EAF			4	1								3
	WPF	2	1	1	1		2	7	31				
2.2.2	AAF	1					7		5	3	2		
	CAF				3	6	7	2	4				
	WEF						6	1	1				
	BMF						6	1	1				
	WPF								5				
2.3.2	AAF	7	19	14	43	21	49	34	122	90	41	40	
	CAF	1	1		2	6	22	5	23	55	22	2	1
	WEF			2	4	23	5	22	22	2	5	7	15
	BMF		1	3	4	26	5	22	22	2	5	6	14
	EAF					4							
	WPF		2	2		4	12	7	42	1		6	1
2.3.4	AAF	3	20	29	110	57	32	15	11	10	1	1	
	CAF		2	4	1	7	6	7	4	2	2		
	WEF		2	2		1		8	8	5	2	16	1
	BMF		1	1		1		8	8	5	2	16	3
	WPF		1	2	2			1	10		1		

AAF, CAF, WEF, BMF, EAF, and WPF represent the East Asian-Australasian Flyway, Central Asian Flyway, West Asian-East African Flyway, Black Sea/Mediterranean Flyway, East Atlantic Flyway, and West Pacific Flyway, respectively. For each clade, flyways with no sequence data sampled are not shown.

**Table 2:** HPAI H5N1 transmission directions of genetic clades.

Clade	Flyway	Number of sample sites	Direction	Concentration	*P*-value
1	East Asian-Australasian Flyway	23	293.94	0.2586	0.001
	Central Asian Flyway	3	277.76	0.9904	0.002
	West Asian-East African Flyway	5	217.89	0.4000	0.001
	Black Sea/Mediterranean Flyway	7	215.29	0.1054	0.388
	East Atlantic Flyway	3	274.00	0.6667	0.007
2.1.3	East Asian-Australasian Flyway	42	71.49	0.4319	0.001
	Central Asian Flyway	7	318.69	0.6147	0.001
	West Asian-East African Flyway	6	331.50	0.4322	0.019
	Black Sea/Mediterranean Flyway	6	216.65	0.9422	0.001
	West Pacific Flyway	12	37.28	0.2711	0.002
2.2.1	East Asian-Australasian Flyway	59	350.34	0.1655	0.001
	Central Asian Flyway	19	258.57	0.1242	0.005
	West Asian-East African Flyway	14	237.58	0.0814	0.171
	Black Sea/Mediterranean Flyway	23	287.90	0.2934	0.001
	West Pacific Flyway	21	329.68	0.4999	0.001
2.2.2	East Asian-Australasian Flyway	10	352.79	0.2973	0.007
	Central Asian Flyway	8	20.58	0.1163	0.264
2.3.2	East Asian-Australasian Flyway	91	39.89	0.1505	0.001
	Central Asian Flyway	27	203.83	0.2671	0.001
	West Asian-East African Flyway	17	52.82	0.1577	0.001
	Black Sea/Mediterranean Flyway	20	347.13	0.1649	0.001
	West Pacific Flyway	25	272.64	0.1891	0.001
2.3.4	East Asian-Australasian Flyway	51	18.93	0.0370	0.049
	Central Asian Flyway	20	279.67	0.2447	0.001
	West Asian-East African Flyway	12	207.38	0.5083	0.001
	Black Sea/Mediterranean Flyway	13	191.97	0.1112	0.062
	West Pacific Flyway	20	279.67	0.2447	0.001
7	East Asian-Australasian Flyway	14	105.94	0.0945	0.238
	Central Asian Flyway	6	234.46	0.3037	0.066
	West Asian-East African Flyway	4	197.55	0.5000	0.002

**Table 3:** Waterfowl seasonal migration directions.

Flyway	Spring migration			*P*-value	Autumn migration			
	Number of sites	Direction	Concentration		Number of sites	Direction	Concentration	*P*-value
East Asian-Australasian Flyway	431	80.56	0.7458	0.001	431	260.56	0.7458	0.001
Central Asian Flyway	564	85.67	0.7184	0.001	564	265.67	0.7184	0.001
West Asian-East African Flyway	382	63.44	0.8272	0.001	382	243.44	0.8272	0.001
Black Sea/Mediterranean Flyway	462	58.04	0.7444	0.001	462	238.04	0.7444	0.001
East Atlantic Flyway	380	63.78	0.6928	0.001	380	243.78	0.6928	0.001
West Pacific Flyway	188	70.21	0.8210	0.001	188	250.21	0.8210	0.001
